# Comparison of Second-Line Chemotherapies for First-Relapsed High-Grade Serous Ovarian Cancer: A Retrospective Study

**DOI:** 10.3390/jcm14196905

**Published:** 2025-09-29

**Authors:** Jeongyun Kim, Se Ik Kim, Dong Hoon Suh, Kidong Kim, Jae Hong No, Yong Beom Kim

**Affiliations:** 1 Department of Obstetrics and Gynecology, Seoul National University College of Medicine, Seoul 03080, Republic of Korea; nero071@snu.ac.kr (J.K.); seikky@naver.com (S.I.K.); sdhwcj@naver.com (D.H.S.); kidong.kim.md@gmail.com (K.K.); 65561@snubh.org (J.H.N.); 2 Department of Obstetrics and Gynecology, Seoul National University Bundang Hospital, Seongnam 13620, Republic of Korea

**Keywords:** chemotherapy, ovarian cancer, platinum resistance, progression-free survival, recurrence

## Abstract

**Background/Objectives**: To compare oncologic outcomes of second-line chemotherapy regimens in relapsed high-grade serous ovarian cancer (HGSOC) by platinum sensitivity. **Methods**: We retrospectively reviewed HGSOC patients treated at two centers (June 2003–December 2020), classified by platinum-free interval (6- and 12-month cut-offs). Outcomes were progression-free survival (PFS, primary) and objective response and disease control rates (secondary). Regimens administered to ≥10% of patients or with favorable outcomes were compared using multivariable Cox analyses. **Results**: Among 468 patients (41.2% sensitive, 32.9% partially sensitive, 25.9% resistant), platinum-sensitive patients were younger (*p* = 0.024), diagnosed earlier, and more likely to undergo primary debulking surgery (both *p* < 0.001), achieving best outcomes after second-line chemotherapy (median PFS 14.8 vs. 10.5 and 5.2 months, *p* < 0.001). In both sensitive groups, the most common regimens were taxane + platinum ± bevacizumab, followed by pegylated liposomal doxorubicin + carboplatin, which was associated with shorter PFS in platinum-sensitive patients (hazard ratio (HR) 1.67, *p* = 0.016). Second-line maintenance with bevacizumab or poly(ADP-ribose) polymerase inhibitors was associated with improved PFS in both groups (*p* < 0.001). In platinum-resistant patients, the omission of bevacizumab (HR 2.01, *p* < 0.001) and a primary treatment history without cytoreduction (HR 4.43, *p* = 0.044) were associated with inferior outcomes. **Conclusions**: In platinum-sensitive patients with a favorable prognosis, taxane + platinum regimens were most commonly used and outperformed PLD + carboplatin. Maintenance therapy also conferred a meaningful benefit. In platinum-resistant disease, bevacizumab use and prior cytoreductive surgery may improve outcomes, underscoring the importance of treatment selection and surgical approach.

## 1. Introduction

Ovarian cancer has the poorest prognosis among gynecologic cancers in both Korea and the United States, despite its relatively low incidence [1,2]. High-grade serous carcinoma, the most common histologic subtype, is usually diagnosed at an advanced stage—stage III in 51% and stage IV in 29% of cases—contributing to its poor prognosis [3]. Recurrence occurs in approximately 25% of patients with early-stage disease and over 80% of those with advanced disease. In such cases, the primary therapeutic goal is disease control rather than complete remission [4]. Accordingly, extensive research has focused on developing and combining agents that target diverse molecular pathways to improve outcomes [5,6]. These include targeted therapies, immune checkpoint inhibitors, antibody–drug conjugates, and hormonal modulators, many of which are currently being evaluated in ongoing clinical trials.

Currently, the standard treatment for recurrent ovarian cancer includes chemotherapy and/or secondary cytoreductive surgery. Prognostic factors, including age, initial International Federation of Gynecology and Obstetrics (FIGO) stage, histology, prior treatment, and recurrence pattern, have been identified [7,8]. Among these, platinum sensitivity—defined by the platinum-free interval (PFI)—is the key determinant in selecting a chemotherapy regimen: platinum-sensitive (PFI ≥ 12 months), partially sensitive (6 ≤ PFI < 12 months), resistant (PFI < 6 months), and refractory (relapse during first-line therapy) [4,9]. For recurrences occurring after six months, carboplatin-based combinations are preferred, whereas non-platinum monotherapy is typically used for early relapse as palliative treatment [10]. More recently, the active use of targeted therapies has been particularly notable. The addition of bevacizumab to conventional chemotherapy has improved progression-free survival (PFS), and the introduction of poly(ADP-ribose) polymerase (PARP) inhibitors has shown promising outcomes in recurrent disease [11,12,13].

The National Comprehensive Cancer Network (NCCN) guidelines present a wide range of therapeutic options for recurrent ovarian cancer, grounded in extensive clinical research to date, which can complicate decision-making and lead to variability in treatment choices among physicians [14]. Furthermore, selecting the optimal regimen requires careful consideration of efficacy, toxicity, and cost.

Given the multiple chemotherapy options for recurrent ovarian cancer—particularly platinum-resistant disease—this study compared oncologic outcomes across commonly used regimens. To enhance clinical relevance, we focused on regimens frequently employed for first-relapsed high-grade serous ovarian cancer (HGSOC), the most common ovarian cancer subtype. Known factors associated with treatment failure were incorporated into the analysis to reduce confounding.

## 2. Materials and Methods

### 2.1. Study Population

This retrospective study analyzed data from patients diagnosed with high-grade serous carcinoma of the ovary, fallopian tube, and primary peritoneum between June 2003 and December 2020 at two tertiary institutions in the Republic of Korea: Seoul National University Bundang Hospital and Seoul National University Hospital. Eligible patients had experienced relapse following platinum-based chemotherapy. The second-line chemotherapy regimens were primarily selected at the physician’s discretion, based on assessments that included disease status, the patient’s treatment history, and insurance coverage. Exclusion criteria were as follows: non-HGSOC histology; another malignancy requiring systemic therapy within three years after ovarian cancer primary treatment; participation in blinded clinical trials; absence of chemotherapy or receipt of only one cycle after recurrence/progression; and first-line regimens lacking platinum agents. Patients who underwent other treatment modalities, such as optimal cytoreductive surgery or pre-/concomitant radiotherapy, were also excluded. This study was approved by the Institutional Review Boards of both institutions (IRB No. B-2404-897-107, H-2409-105-1573). The requirement for informed consent was waived owing to its retrospective design and use of de-identified data.

### 2.2. Outcomes

Patients were classified according to the PFI following their last chemotherapy, using cutoffs of 6 and 12 months. However, in the case of refractory disease, we combined it with resistant disease, considering that their chemotherapy regimens are essentially the same. This study aimed to compare PFS after second-line chemotherapy stratified by PFI-based groups. The primary outcome, PFS, was defined as the interval from initiation of second-line chemotherapy to second recurrence or progression, or, if not documented, to initiation of third-line therapy, last follow-up, or death, whichever occurred first. Secondary outcomes included objective response rate (ORR) and disease control rate (DCR). ORR was defined as the proportion of patients achieving complete response (CR) or partial response (PR), and DCR as those with CR, PR, or stable disease. Treatment response was evaluated according to Response Evaluation Criteria in Solid Tumors version 1.1 or CA125-based Gynecologic Cancer Intergroup criteria [15,16]. Patients visited their physicians on a predetermined schedule or when specific symptoms arose. They received necessary imaging—primarily abdominopelvic computed tomography—and CA-125 testing, following current guidelines with adjustments based on clinical context. Tumor response was assessed through a combination of imaging and serum tests. In cases of discordance, physicians conducted a comprehensive evaluation, considering the clinical situation and patient symptoms, and performed diagnostic interventions when necessary.

### 2.3. Statistical Analysis

Clinicopathological variables were compared using one-way ANOVA, the Kruskal–Wallis test, the independent *t*-test, or the Mann–Whitney U test for continuous variables, as appropriate based on parametric testing. For categorical variables, the chi-square test or Fisher’s exact test was applied, as applicable. Post hoc pairwise comparisons were performed with Bonferroni adjustment.

Treatment regimens administered to 10% or more of patients or showing favorable outcomes were compared using the Kaplan–Meier analysis and log-rank tests, with median PFS (mPFS) estimates and 95% confidence intervals (CIs) calculated. A Cox proportional hazards model was constructed to evaluate factors associated with PFS, with hazard ratios (HRs) and 95% CIs calculated. Age, year of diagnosis, initial stage, primary treatment modality, extent of primary cytoreduction, and maintenance therapy after secondary treatment were assessed in the univariable analysis, and variables with *p* < 0.1 were included in the multivariable model. If certain regimens required further evaluation (e.g., showing results contradictory to previous findings), their characteristics were compared, and the Cox model was further adjusted to include PFI and first-line maintenance therapy, in addition to the aforementioned factors, to control for confounding. Due to expected heterogeneity among variables, inverse probability of treatment weighting (IPTW) was applied to variables showing significant differences in baseline characteristics. Subsequently, a weighted Cox proportional hazards model for PFS was constructed to analyze recurrence risk further.

Statistical analyses were performed using SPSS version 29.0 (IBM Corp., Armonk, NY, USA). The ORR and DCR, along with their 95% CIs based on the exact binomial (Clopper–Pearson) method, as well as survival curves and IPTW, were generated using R software (version 4.5.1; R Foundation for Statistical Computing, Vienna, Austria), utilizing the binom, survival, survminer, WeightIt, cobalt, and survey packages [17,18,19]. Two-tailed *p*-values < 0.05 were considered statistically significant.

## 3. Results

A total of 468 patients were included in the analysis, comprising 163 (34.8%) from Seoul National University Bundang Hospital and 305 (65.2%) from Seoul National University Hospital: 193 (41.2%) platinum-sensitive (PFI ≥ 12 months, median 18.1 months), 154 (32.9%) partially sensitive (6 ≤ PFI < 12 months, median 7.9 months), and 121 (25.9%) platinum-resistant (PFI < 6 months, median 3.5 months). Baseline characteristics stratified by platinum sensitivity are summarized in Table 1. The three groups differed significantly in age, initial stage, and primary treatment method (*p* = 0.024, <0.001, and <0.001, respectively), with post hoc results as follows: Platinum-sensitive patients were significantly younger than platinum-resistant patients (mean age 56.0 years ± 10.4 vs. 59.0 years ± 10.7, *p* = 0.039). Compared to the partially sensitive group, they had a higher proportion of early-stage disease (12.4% vs. 2.6%, *p* < 0.001), more frequent primary debulking surgery (PDS, 65.8% vs. 46.1%), and lower rates of interval debulking surgery (IDS, 33.2% vs. 50.0%) and no surgery (1.0% vs. 3.9%) (*p* < 0.001). Although not statistically significant, more were diagnosed between 2003 and 2014 (50.8% vs. 38.6% and 47.1%, *p* = 0.243), achieved no residual disease status (60.7% vs. 52.0% and 49.6%, *p* = 0.272), and received maintenance therapy after first-line chemotherapy (14.0% vs. 7.8% and 4.1%, *p* = 0.216). All patients received first-line chemotherapy with a taxane (paclitaxel or docetaxel) and a platinum agent (carboplatin or cisplatin), with or without bevacizumab, except one who received topotecan plus carboplatin due to paclitaxel-related toxicity.

**Figure 1:**
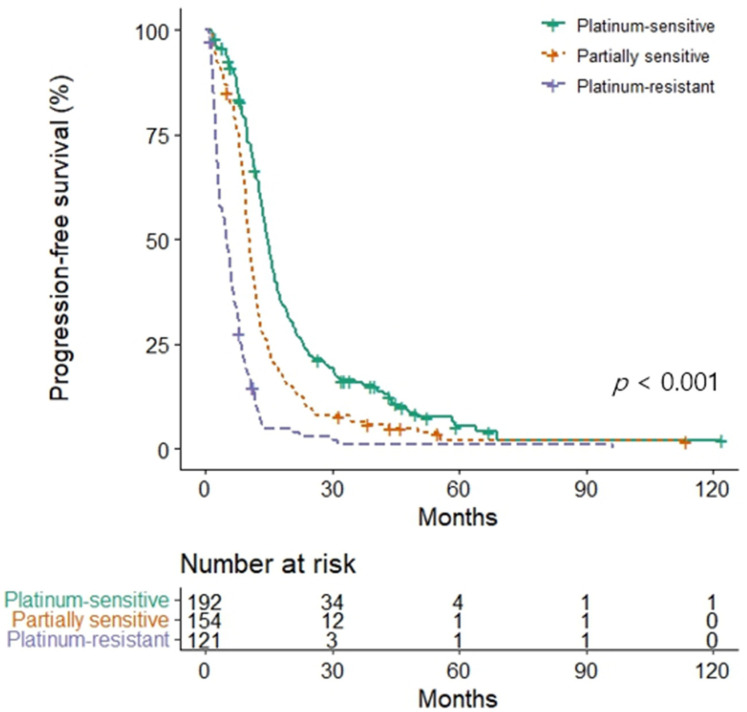
Progression-free survival following second-line chemotherapy based on prior platinum-free intervals.

Among them, 432 patients (92.3%) experienced a second recurrence. A weighted Cox model adjusting for initial stage, primary treatment method, and age showed excellent covariate balance (maximum standardized mean difference < 0.017) and good predictive performance (concordance index = 0.674). PFI was independently associated with recurrence: compared to the platinum-sensitive group, the partially sensitive group had a higher risk (HR 1.53, 95% CI 1.23–1.90, *p* < 0.001), and the platinum-resistant group had an even greater risk (HR 3.65; 95% CI, 2.47–5.41, *p* < 0.001). After second-line chemotherapy, oncologic outcomes across the three groups—platinum-sensitive, partially sensitive, and platinum-resistant—were as follows: mPFS (14.8 vs. 10.5 vs. 5.2 months; Figure 1), ORR (81.8% vs. 67.3% vs. 29.2%), and DCR (94.8% vs. 89.5% vs. 62.5%) (all *p* < 0.001).

With a median follow-up of 10.2 months, responses to second-line chemotherapies are detailed in Table 2. Among platinum-sensitive and partially sensitive patients, the most frequently used regimens were taxane + platinum ± bevacizumab and pegylated liposomal doxorubicin (PLD) + carboplatin. Although based on small samples (*n* = 6 for sensitive and *n* = 5 for partially sensitive), gemcitabine + platinum + bevacizumab demonstrated promising efficacy, with mPFS of 15.5 and 15.0 months, respectively, and 100% ORR and DCR in both groups. Outcomes were inferior when bevacizumab was omitted. In platinum-resistant patients, the most commonly used regimens were PLD + bevacizumab, belotecan/topotecan monotherapy, and belotecan/topotecan + cisplatin. Across regimens, the addition of bevacizumab was consistently associated with improved outcomes: taxane + platinum + bevacizumab vs. taxane + platinum (mPFS 11.6 vs. 4.6 months), PLD + bevacizumab vs. PLD alone (mPFS 6.2 vs. 2.6 months), and topotecan + bevacizumab vs. belotecan/topotecan alone (mPFS 9.6 vs. 4.9 months).

**Figure 2:**
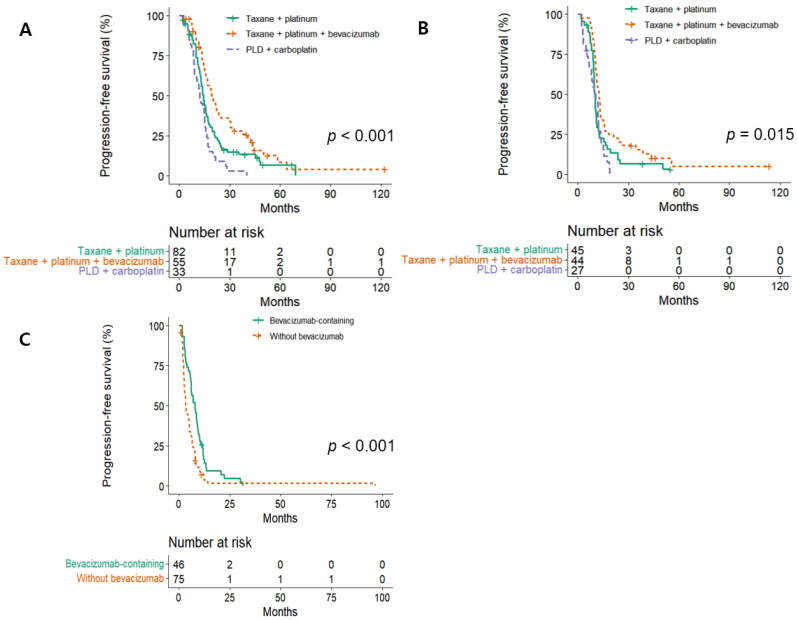
Progression-free survival following second-line chemotherapy regimens commonly used (≥10% of patients) in platinum-sensitive and partially sensitive groups, and those associated with favorable outcomes in platinum-resistant patients (in this case, bevacizumab): (**A**) platinum-sensitive, (**B**) partially sensitive, and (**C**) resistant. Abbreviations: PLD, pegylated liposomal doxorubicin. Taxane refers to either paclitaxel or docetaxel, while platinum refers to either carboplatin or cisplatin.

In both sensitive groups, three chemotherapy regimens (each used in at least 10% of patients) were compared within each PFI category (Figure 2). In the platinum-resistant group, comparisons were based on bevacizumab use, given its evident therapeutic benefit. To adjust for confounders, multivariable Cox models were constructed (Table 3), including variables significant in univariable analysis: second-line maintenance therapy for both sensitive groups, and primary treatment modality (timing of debulking surgery) for the resistant group. In the platinum-sensitive group, PLD + carboplatin was associated with inferior outcomes compared to taxane + platinum (HR 1.67, 95% CI 1.10–2.54, *p* = 0.016), a difference not observed in the partially sensitive group. Conversely, maintenance therapy with PARP inhibitors significantly improved outcomes (platinum-sensitive: HR 0.36, 95% CI 0.22–0.59, *p* < 0.001; partially sensitive: HR 0.34, 95% CI 0.17–0.68, *p* = 0.002), as did bevacizumab maintenance (platinum-sensitive: HR 0.44, 95% CI 0.31–0.63; partially sensitive: HR 0.43, 95% CI 0.28–0.66; both *p* < 0.001).

Among platinum-resistant patients, regimens without bevacizumab were associated with poorer outcomes (HR 2.01, 95% CI 1.36–2.97, *p* < 0.001). A primary treatment history without debulking surgery was also significantly associated with inferior outcomes (HR 4.43, 95% CI 1.04–18.90, *p* = 0.044). Both sensitive groups showed a trend toward decreased PFS without surgery, but the differences were not statistically significant (mPFS for the platinum-sensitive group: 15.2, 15.1, and 8.8 months for PDS, IDS, and no surgery, respectively; mPFS for the partially sensitive group: 12.1, 10.7, and 10.9 months, respectively).

Additional variables that did not reach statistical significance in univariable Cox regression are summarized in Table S1.

When focusing on cytotoxic agents used as second-line chemotherapy, only the comparison between taxane + platinum ± bevacizumab and PLD + carboplatin showed a significant difference in the platinum-sensitive patients. Their baseline characteristics were further analyzed (Table 4). Patients receiving taxane + platinum had a longer median PFI after first-line chemotherapy (21.1 vs. 15.3 months, *p* = 0.005), were more often diagnosed in earlier years (2003–2014: 53.3% vs. 24.2%, *p* = 0.003), and underwent different primary treatment modalities (PDS: 70.8% vs. 54.5%; IDS: 29.2% vs. 42.4%; no surgery: 0.0% vs. 3.0%; *p* = 0.045). Residual disease extent after cytoreductive surgery (PDS or IDS) did not differ significantly (*p* = 0.320), but suboptimal cytoreduction was less frequent with the taxane + platinum group (11.7% vs. 21.9%). Both first- and second-line maintenance therapies showed significantly different distributions (*p* < 0.001). The PLD + carboplatin group received more first-line maintenance treatment, particularly bevacizumab (27.3% vs. 4.4% in the taxane + platinum group), and second-line PARP inhibitors (18.2% vs. 13.9%).

For platinum-sensitive patients treated with either taxane + platinum or PLD + carboplatin, univariable and multivariable Cox regression models (Table S2) identified chemotherapy regimen and second-line maintenance therapy as the only statistically significant factors. After adjustment, PLD + carboplatin was associated with a higher risk for recurrence compared to taxane + platinum (HR 1.67, 95% CI 1.10–2.53, *p* = 0.015). Conversely, second-line maintenance with bevacizumab (HR 0.47, 95% CI 0.31–0.70, *p* < 0.001) or PARP inhibitors (HR 0.34, 95% CI 0.20–0.57, *p* < 0.001) was associated with favorable outcomes. A weighted Cox model, adjusted for PFI, year of diagnosis, primary treatment modality, and both first- and second-line maintenance therapies, confirmed the increased recurrence risk with PLD + carboplatin versus taxane + platinum (HR 1.90, 95% CI 1.25–2.89, *p* = 0.003). Covariate balance was acceptable except for some imbalance in second-line bevacizumab maintenance (standardized mean difference = 0.28). The model’s predictive ability was modest (concordance index = 0.572).

## 4. Discussion

This retrospective study evaluated real-world practices and outcomes in patients with recurrent or persistent HGSOC by analyzing the regimens used in clinical settings. Platinum-sensitive patients, who had the best prognosis after second-line chemotherapy, responded favorably to platinum-based regimens, particularly their first-line treatment (taxanes + platinum ± bevacizumab). Partially sensitive patients showed similar trends. In both sensitive groups, second-line maintenance therapy with PARP inhibitors or bevacizumab was associated with improved PFS, regardless of the chemotherapy regimen. These findings align with previous clinical trials and support the use of targeted therapies in recurrent disease.

Since the introduction of a PARP inhibitor, olaparib, as first-line maintenance in 2018 (following its initial approval for recurrent disease in 2014), these agents have reshaped the treatment landscape of ovarian cancer, with proven efficacy in both newly diagnosed and recurrent disease [13]. However, as our cohort included patients initially diagnosed between 2003 and 2020, the uptake of first-line PARP inhibitor maintenance was limited (1.7%). As a result, its impact could not be meaningfully assessed and was excluded from the multivariable analysis, though it was included in the subset analysis. In second-line treatment, PARP inhibitor maintenance was consistently and significantly associated with longer PFS in both platinum-sensitive and partially sensitive patients.

The use of PARP inhibitors is closely linked to patients’ genetic profiles, which also influence chemotherapy response and prognosis [20]. Approximately 50% of HGSOCs are estimated to exhibit a high degree of genomic instability due to homologous recombination deficiency (HRD), commonly associated with and well-studied in the context of BRCA1/2 mutations. With PARP inhibitors now approved and widely recommended based on BRCA1/2 mutation status and HRD, genetic testing has become increasingly important in clinical practice. However, since about 72% of patients in this study were diagnosed before 2018, further research is needed to evaluate current cytotoxic regimens and the role of genetic profiling in treatment selection and outcomes.

Although paclitaxel + carboplatin remains the standard first-line chemotherapy for ovarian cancer, paclitaxel can be replaced by docetaxel, and carboplatin by cisplatin, depending on the patient’s condition and drug toxicity [21,22]. Such adjustments may also occur even during treatment, with physicians opting for regimens with similar mechanisms but different toxicity profiles. This approach likewise applies to belotecan and topotecan [23]. Accordingly, taxanes (paclitaxel and docetaxel), platinum agents (carboplatin and cisplatin), and topoisomerase I inhibitors (belotecan and topotecan) were grouped for efficacy evaluation and comparison. While the exact scheduling, including dosing and timing, may influence oncologic outcomes, we aimed to reflect real-world clinical practices, where such factors are often adjusted based on patient condition, toxicity, or hypersensitivity.

Notably, the PLD + carboplatin regimen showed inferior outcomes compared to the taxane + platinum regimen in platinum-sensitive recurrent or persistent HGSOC patients in our cohort. This contrasts with the findings from the CALYPSO trial, a randomized phase III study, which reported superior PFS and efficacy with this regimen compared to paclitaxel + carboplatin [24,25,26]. Due to our study’s retrospective design and small sample size, these results should be interpreted with caution. Additionally, the taxane + platinum group exhibited significantly more favorable baseline characteristics, including longer PFI and a higher rate of PDS. Although not statistically significant, they also tended to be younger and less likely to have undergone suboptimal surgery. However, these traits did not translate into significant outcomes in the Cox regression model. Only the chemotherapy regimen and maintenance therapy showed significant associations with PFS.

Certain widely used treatment approaches continue to have controversial efficacy and risks. In particular, concerns about increased platinum resistance following IDS have been consistently raised, leading to ongoing debate due to conflicting study results [27,28,29]. Moreover, a Japanese trial demonstrated that weekly paclitaxel combined with triweekly carboplatin improved PFS and overall survival compared to the conventional triweekly regimen [30]. However, a European multicenter trial and a study including patients from the United States, Canada, and the Republic of Korea—where 84% of patients received bevacizumab—concluded that weekly paclitaxel did not provide benefits in PFS [31,32]. The findings of our study should be interpreted in light of these considerations. Nonetheless, after adjusting for potential factors that could lead to treatment failure, the chemotherapy regimen remained significantly associated with PFS.

Also, unlike the CALYPSO trial, our cohort was limited to patients with first relapse and high-grade serous histology, excluding those who underwent secondary cytoreduction. In addition to these baseline differences, treatment dose and scheduling were not considered; only regimen types were analyzed. Notably, CALYPSO remains the only randomized trial to date that has demonstrated superior outcomes for PLD + carboplatin compared to paclitaxel + carboplatin in the pre-maintenance treatment era. Recent data reflecting evolving treatment strategies, including maintenance therapy, should be used further to investigate the efficacy of previously used chemotherapy regimens.

While most HGSOCs are initially platinum-sensitive, they eventually develop resistance over time, resulting in a poor prognosis [33]. This pattern of acquired resistance is not limited to platinum agents but is also observed with other initially effective therapies, and currently, no validated method exists for predicting resistance [34]. To reduce the impact of anticipated chemotherapy resistance and cumulative toxicity, we focused our cohort on patients with first-relapsed HGSOC, excluding those who were heavily pretreated. In addition, although ORR and DCR are commonly used to assess chemotherapy response, these metrics may not fully reflect long-term efficacy. For example, one platinum-resistant patient treated with oral etoposide achieved a PFS of 8.3 months, despite only achieving stable disease before eventual progression. In this recurrent/persistent and predominantly palliative setting, PFS—used as the primary outcome in our study—may therefore serve as a more reliable indicator of treatment efficacy than ORR or DCR.

Meanwhile, despite having the poorest prognosis, platinum-resistant patients demonstrated improved outcomes with bevacizumab-containing regimens. Conversely, primary treatment without cytoreduction was associated with shorter PFS, which may reflect either the biological effects of surgery or the poor systemic condition of patients who were unsuitable for aggressive cytoreduction.

Bevacizumab, an anti-VEGF antibody, has demonstrated benefits across sensitivity groups. Its use, either as maintenance in sensitive disease or in combination with cytotoxic agents in resistant cases, was associated with improved outcomes, consistent with previous studies [11,35]. Prior to the introduction of bevacizumab, platinum-resistant or refractory patients were typically managed with monotherapy, which yielded low response rates of 10–20% and served mainly palliative purposes, aiming to reduce toxicity, as no combination therapy had proven superior [36]. Notably, bevacizumab appears to be unaffected by resistance mechanisms to conventional chemotherapeutic agents or PARP inhibitors and is anticipated to play a pivotal role in managing chemo-refractory and recurrent ovarian cancer [37].

In platinum-resistant patients, the gemcitabine + platinum regimen showed better responses than gemcitabine monotherapy, suggesting that platinum retreatment may still be feasible in specific cases. Additionally, oral etoposide demonstrated an acceptable PFS of 8.3 months in our cohort, consistent with previous studies, and may be considered a palliative treatment option, particularly given the convenience of enteral administration [38].

This study has several notable limitations. First, the study is based on data from two tertiary centers in the Republic of Korea with a small sample size, which limits the generalizability of the findings. Although we focused on a real-life clinical scenario, we did not account for external factors (e.g., insurance coverage and healthcare infrastructure) or patient-specific variables (e.g., race, genetic profiles, frailty, inflammatory markers, tumor burden, and prior treatment-related toxicity). These factors can influence both clinical decision-making and patient outcomes, and may vary across regions, further limiting the generalizability of the results. Clinical decisions are multi-factorial, involving patient comorbidities, prior treatment history and toxicity, as well as oncologic outcomes. Therefore, focusing solely on oncologic outcomes is inherently limiting. Additionally, while the clinical reasoning process, which involves various factors including physician preference, is valuable for documentation and analysis, it could not be accurately evaluated due to the retrospective nature of this study.

Moreover, the retrospective design may introduce selection and information biases, complicating the distinction between recurrent and persistent disease. Accurate pre-treatment assessment of tumor burden could improve evaluation of drug resistance and tumor response [39]. Also, the exact treatment regimens, including drug dosages, likely varied over time and were tailored to each patient’s tolerability. While these factors are important for accurate comparisons and may influence outcomes, this study did not perform an in-depth analysis to facilitate a straightforward comparison among various treatment strategies. Genetic factors such as BRCA mutation status and HRD, which may influence oncologic outcomes, were not adequately described since the cohort mainly spans the pre-PARP inhibitor era.

Finally, broad comparisons require careful consideration of each regimen’s specific characteristics for accurate and nuanced interpretation. The heterogeneity among cancer patients in this study should also be taken into account, regardless of the statistical tests performed. This approach, which accounts for baseline characteristics, may help identify patient populations more likely to benefit from specific chemotherapy regimens, aligning with the goals of the current precision medicine era. To better reflect real-world scenarios, we limited further analysis to regimens that were either commonly used or yielded results contrary to those reported in previous studies. For platinum-resistant patients, classification was based on bevacizumab use, given the diversity of regimens and the lack of proven superiority among them. However, among the less commonly used therapies, some showed favorable outcomes, highlighting the need for further research to identify appropriate target populations for these treatments.

Despite these limitations, this study aimed to provide real-world insights into clinical decision-making, particularly regarding therapeutic choices following recent recommendations and findings from key clinical trials. The treatment outcomes can be considered a reflection of various factors that may influence the course of therapy, including treatment-related toxicities.

## 5. Conclusions

Platinum sensitivity is a well-established prognostic factor in recurrent or persistent HGSOC. In platinum-sensitive patients, commonly used taxane–platinum combinations, with or without bevacizumab, showed superior outcomes compared to PLD–carboplatin. While diverse chemotherapy options remain important for patients with comorbidities or treatment-related toxicities, treatment decisions should be more precise and personalized, supported by robust evidence and a precision medicine approach. Targeted therapies, including bevacizumab and PARP inhibitors, should be actively incorporated due to their proven efficacy. For platinum-resistant patients, who generally have the poorest prognosis, bevacizumab-containing regimens and possibly primary cytoreductive surgery may offer benefit. Further research is needed to improve outcomes in platinum-resistant disease, particularly among those without prior primary debulking surgery. As clinical practices evolve, rigorous comparisons of widely used regimens are essential to guide treatment strategies. Future research should integrate clinical variables to better account for patient-specific factors, thereby optimizing both oncologic and non-oncologic outcomes.

## Figures and Tables

**Table 1:** Clinical characteristics of patients who received second-line chemotherapy for recurrent/persistent high-grade serous ovarian cancer, based on platinum sensitivity.

Characteristics	Platinum Sensitivity			*p* Value
	Sensitive (*n* = 193, 41.2%)	Partially Sensitive (*n* = 154, 32.9%)	Resistant (*n* = 121, 25.9%)	
PFI (months), median (range)	18.1 (12.1–164.4)	7.9 (6.0–12.0)	3.5 (0.3–5.98)	
Age at diagnosis (years), mean ±SD	56.0 ± 10.4	58.3 ± 10.2	59.0 ± 10.7	0.024
<60	125 (64.8)	87 (56.5)	61 (50.4)	0.036
≥60	68 (35.2)	67 (43.5)	60 (49.6)	
Year of diagnosis, *n* (%)				0.243
2003–2014	98 (50.8)	59 (38.6)	57 (47.1)	
2015–2017	46 (23.8)	45 (29.4)	33 (27.3)	
2018-2020	49 (25.4)	49 (32.0)	31 (25.6)	
Initial FIGO Stage, *n* (%)				<0.001
Early (I–II)	24 (12.4)	4 (2.6)	4 (3.3)	
Advanced (III–IV)	169 (87.6)	150 (97.4)	117 (96.7)	
Primary treatment, *n* (%)				<0.001
Primary debulking surgery	127 (65.8)	71 (46.1)	50 (41.3)	
Interval debulking surgery	64 (33.2)	77 (50.0)	69 (57.0)	
No surgery	2 (1.0)	6 (3.9)	2 (1.7)	
Primary cytoreduction extent *, *n* (%)				0.272
No residual	116 (60.7)	77 (52.0)	59 (49.6)	
Optimal (residual tumor < 1 cm)	42 (22.0)	35 (23.6)	35 (29.4)	
Suboptimal	26 (13.6)	30 (20.3)	23 (19.3)	
Unknown	7 (3.7)	6 (4.1)	2 (1.7)	
First-line maintenance, *n* (%)				0.216
None	166 (86.0)	142 (92.2)	116 (95.9)	
Bevacizumab	17 (8.8)	9 (5.8)	4 (3.3)	
PARP inhibitors	5 (2.6)	2 (1.3)	1 (0.8)	
Paclitaxel	4 (2.1)	1 (0.6)	0 (0.0)	
Oral etoposide	1 (0.5)	0 (0.0)	0 (0.0)	
Second-line chemotherapy				
PFS (months), median (95% CI)	14.8 (13.5–16.1) ^†^	10.5 (9.7–11.4)	5.2 (3.8–6.5)	<0.001
ORR, % (95% CI) ^‡^	81.8 (75.6–87.0)	67.3 (59.3–74.7)	29.2 (21.2–38.2)	<0.001
DCR, % (95% CI) ^‡^	94.8 (90.6–97.5)	89.5 (83.6–93.9)	62.5 (53.2–71.2)	<0.001

Abbreviations: CI, confidence interval; DCR, disease control rate; FIGO, International Federation of Gynecology and Obstetrics; ORR, objective response rate; PARP, poly(adenosine diphosphate-ribose) polymerase; PFI, platinum-free interval; PFS, progression-free survival; SD, standard deviation. * Only patients undergoing primary or interval debulking surgery during primary treatment were analyzed. ^†^ One patient’s progression-free survival was not evaluable due to loss to follow-up. ^‡^ In each group, one patient was not evaluable for response due to a lack of post-treatment assessment after at least two cycles of chemotherapy.

**Table 2:** Responses to second-line chemotherapy based on platinum sensitivity.

Regimen	Platinum Sensitivity											
	Sensitive (*n* = 193, 41.2%)				Partially Sensitive (*n* = 154, 32.9%)				Resistant (*n* = 121, 25.9%)			
	*n* (%)	ORR	DCR	PFS *	*n* (%)	ORR	DCR	PFS *	*n* (%)	ORR	DCR	PFS *
Taxane + platinum ^†^	**82 (42.5)**	**86.6**	**95.1**	**14.3**	**45 (29.2)**	**79.5 ^‡^**	**90.9 ^‡^**	**10.1**	4 (3.3)	50	75	4.6
Taxane + platinum + bevacizumab ^†^	**55 (28.5)**	**92.7**	**98.2**	**19.3**	**44 (28.6)**	**77.3**	**97.7**	**12.5**	**2 (1.7)**	**100**	**100**	**11.6**
Paclitaxel + bevacizumab	0 (0.0)				0				**2 (1.7)**	**0**	**50**	**4.9**
PLD	0 (0.0)				0				10 (8.3)	0 ^‡^	11.1 ^‡^	2.6
PLD + carboplatin	**33 (17.1)**	**63.6**	**87.9**	**12.3**	**27 (17.5)**	**48.1**	**81.5**	**10.7**	1 (0.8)	100	100	8.1
PLD + bevacizumab	0 (0.0)				3 (1.9)	33.3	33.3	5.8	**32 (26.4)**	**34.4**	**68.8**	**6.2**
Gemcitabine	0(0.0)				0 (0.0)				2 (1.7)	0	0	2.7
Gemcitabine + platinum ^†^	10 (5.2)	66.7 ^‡^	100 ^‡^	13.8 ^§^	12 (7.8)	66.7	91.7	9.8	3 (2.5)	66.7	100	6.6
Gemcitabine + platinum + bevacizumab ^†^	6 (3.1)	100	100	15.5	5 (3.2)	100	100	15	0 (0.0)			
Belotecan/topotecan	3 (1.6)	0	100	6.6	12 (7.8)	33.3	83.3	5	33 (27.3)	21.2	63.6	4.9
Belotecan/topotecan + cisplatin	2 (1.0)	100	100	14.2	5 (3.2)	60	80	12.2	18 (14.9)	22.2	55.6	3.4
Topotecan + bevacizumab	0 (0.0)				0				**10 (8.3)**	**60**	**100**	**9.6**
Cyclophosphamide + cisplatin ± doxorubicin	0 (0.0)				0				2 (1.7)	0	0	2
Carboplatin	0 (0.0)				0				1 (0.8)	0	0	1.7
Oral etoposide	2 (1.0)	0	50	4.6	0				1 (0.8)	0	100	8.3
Pembrolizumab	0 (0.0)				1 (0.6)	0	100	5.4	0 (0.0)			
Second-line maintenance												
None	118 (61.1)				93 (60.4)				119 (98.3)			
Bevacizumab	50 (25.9)				44 (28.6)				2 (1.7)			
PARP inhibitors	25 (13.0)				15 (9.7)				0 (0.0)			
Bevacizumab + PARP inhibitors	0 (0.0)				1 (0.6)				0 (0.0)			
Paclitaxel	0 (0.0)				1 (0.6)				0 (0.0)			

Regimens that were further evaluated are bolded: in the platinum-sensitive and partially sensitive groups, those administered to ≥10% of patients; in the platinum-resistant group, bevacizumab-containing regimens versus those without bevacizumab. Abbreviations: DCR, disease control rate; ORR, objective response rate; PARP, poly(adenosine diphosphate-ribose) polymerase; PFS, progression-free survival; PLD, pegylated liposomal doxorubicin. * Median (months). ^†^ Taxane refers to either paclitaxel or docetaxel, while platinum refers to either carboplatin or cisplatin. ^‡^ One patient’s response was not evaluable due to the absence of assessment after at least two chemotherapy cycles. ^§^ One patient’s progression-free survival was not evaluable due to loss to follow-up.

**Table 3:** Univariable and multivariable Cox regression analyses of progression-free survival within each platinum sensitivity group (In sensitive/partially sensitive groups, patients receiving regimens administered to ≥10% of the group were included; platinum-resistant regimens grouped by bevacizumab use).

Variable	*n* (%)	PFS (95% CI) ^†^	Univariable		Multivariable	
			HR (95% CI)	*p* Value	HR (95% CI)	*p* Value
**Platinum-sensitive (*n* = 170)**						
Chemotherapy regimen						
Taxane + platinum ^‡^	82 (48.2)	14.3 (12.7–15.8)	Reference		Reference	
Taxane + platinum + bevacizumab ^‡^	55 (32.4)	19.3 (13.6–25.0)	0.65 (0.44–0.94)	0.023	0.999 (0.43–2.31)	0.998
PLD + carboplatin	33 (19.4)	12.3 (8.8–15.8)	1.66 (1.09–2.53)	0.017	1.67 (1.10–2.54)	0.016 *
Second-line maintenance						
None	99 (58.2)	12.8 (11.1–14.6)	Reference		Reference	
Bevacizumab	46 (27.1)	19.3 (12.9–25.6)	0.42 (0.28–0.62)	<0.001	0.44 (0.31–0.63)	<0.001 *
PARP inhibitors	25 (14.7)	21.3 (18.8–23.8)	0.34 (0.20–0.57)	<0.001	0.36 (0.22–0.59)	<0.001 *
**Partially sensitive (*n* = 116)**						
Chemotherapy regimen						
Taxane + platinum ^‡^	45 (38.8)	10.1 (9.5–10.7)	Reference		Reference	
Taxane + platinum + bevacizumab ^‡^	44 (37.9)	12.5 (10.7–14.4)	0.63 (0.41–0.97)	0.038	0.96 (0.32–2.92)	0.948
PLD + carboplatin	27 (23.3)	10.7 (6.4–14.9)	1.26 (0.77–2.08)	0.356	-	-
Second-line maintenance						
None	61 (52.6)	9.8 (9.2–10.3)	Reference		Reference	
Bevacizumab	40 (34.5)	12.5 (10.8–14.2)	0.43 (0.28–0.66)	<0.001	0.43 (0.28–0.66)	<0.001 *
PARP inhibitors	13 (11.2)	12.3 (1.7–22.8)	0.34 (0.17–0.68)	0.002	0.34 (0.17–0.68)	0.002 *
Bevacizumab + PARP inhibitors	1 (0.9)	34.3 (NA)	0.23 (0.03–1.69)	0.149	-	-
Paclitaxel	1 (0.9)	10.3 (NA)	1.16 (0.16–8.40)	0.887	-	-
**Platinum-resistant (*n* = 121)**						
Chemotherapy regimen						
Bevacizumab-containing regimen	46 (38.0)	7.8 (5.5–10.0)	Reference		Reference	
Without bevacizumab	75 (62.0)	3.4 (2.6–4.3)	1.91 (1.30–2.79)	<0.001	2.01 (1.36–2.97)	<0.001 *
Primary treatment						
Primary debulking surgery	50 (41.3)	5.1 (3.8–6.5)	Reference		Reference	
Interval debulking surgery	69 (57.0)	5.7 (3.7–7.8)	1.22 (0.84–1.79)	0.297	-	-
No surgery	2 (1.7)	1.8 (NA)	5.21 (1.22–22.20)	0.026	4.43 (1.04–18.90)	0.044 *

Abbreviations: CI, confidence interval; HR, hazard ratio; NA, not applicable; PARP, poly(adenosine diphosphate-ribose) polymerase; PFS, progression-free survival; PLD, pegylated liposomal doxorubicin. * Significantly different. ^†^ Median (months). ^‡^ Taxane refers to either paclitaxel or docetaxel, while platinum refers to either carboplatin or cisplatin.

**Table 4:** Clinical characteristics of platinum-sensitive patients who received taxane plus platinum or pegylated liposomal doxorubicin (PLD) plus carboplatin as second-line chemotherapy.

Characteristics	Taxane + Platinum ^†^	PLD + Carboplatin	*p* Value
	(*n* = 137, 71.0% ^‡^)	(*n* = 33, 17.1% ^‡^)	
PFI (months), median (range) after first-line chemotherapy	21.1 (12.1–164.4)	15.3 (12.2–42.5)	0.005 *
Age at diagnosis (years), mean ± SD	55.5 ± 9.9	59.0 ± 11.6	0.06
Year of diagnosis, *n* (%)			0.003 *
2003–2014	73 (53.3)	8 (24.2)	
2015–2017	28 (20.4)	15 (45.5)	
2018–2020	36 (26.3)	10 (30.3)	
Initial FIGO stage, *n* (%)			0.787
Early (I–II)	19 (13.9)	5 (15.2)	
Advanced (III–IV)	118 (86.1)	28 (84.8)	
Primary treatment, *n* (%)			0.045 *
Primary debulking surgery	97 (70.8)	18 (54.5)	
Interval debulking surgery	40 (29.2)	14 (42.4)	
No surgery	0 (0.0)	1 (3.0)	
Primary cytoreduction extent ^§^, *n* (%)			0.32
No residual	84 (61.3)	20 (62.5)	
Optimal (residual tumor < 1 cm)	31 (22.6)	5 (15.6)	
Suboptimal (residual tumor ≥ 1 cm)	16 (11.7)	7 (21.9)	
Unknown	6 (4.4)	0 (0.0)	
First-line maintenance, *n* (%)			<0.001 *
None	126 (92.0)	23 (69.7)	
Bevacizumab	6 (4.4)	9 (27.3)	
PARP inhibitors	4 (2.9)	1 (3.0)	
Paclitaxel	1 (0.7)	0 (0.0)	
Second-line maintenance, *n* (%)			
None	72 (52.6)	27 (81.8)	<0.001 *
Bevacizumab	46 (33.6)	0 (0.0)	
PARP inhibitors	19 (13.9)	6 (18.2)	

Abbreviations: FIGO, International Federation of Gynecology and Obstetrics; PARP, poly(adenosine diphosphate-ribose) polymerase; PFI, platinum-free interval; SD, standard deviation. * Significantly different. ^†^ Taxane refers to either paclitaxel or docetaxel, while platinum refers to either carboplatin or cisplatin. ^‡^ Percentages refer to the proportion of patients within the platinum-sensitive group only. ^§^ Only patients undergoing primary or interval debulking surgery during primary treatment were analyzed.

## Data Availability

Data supporting the results of this manuscript are available from the corresponding author upon reasonable request.

## Supplementary Materials

The following supporting information can be downloaded at: https://www.mdpi.com/article/10.3390/jcm14196905/s1, Table S1. Univariable Cox regression analyses of progression-free survival showing non-significant results within each platinum sensitivity group (In sensitive/partially sensitive groups, patients receiving regimens administered to ≥10% of the group were included); Table S2. Univariable and multivariable Cox regression analyses of progression-free survival for platinum-sensitive patients who received taxane plus platinum or pegylated liposomal doxorubicin (PLD) plus carboplatin as second-line chemotherapy.

## Abbreviations

The following abbreviations are used in this manuscript:

HGSOC	high-grade serous ovarian cancer
PFS	progression-free survival
PLD	pegylated liposomal doxorubicin
HR	hazard ratio
CI	confidence interval
FIGO	International Federation of Gynecology and Obstetrics
PFI	platinum-free interval
PARP	poly(ADP-ribose) polymerase
NCCN	National Comprehensive Cancer Network
ORR	objective response rate
DCR	disease control rate
CR	complete response
PR	partial response
SD	standard deviation
NA	not applicable
