# Supplementary material for: Comparison of Second-Line Chemotherapies for First-Relapsed High-Grade Serous Ovarian Cancer: A Retrospective Study
Source: J Clin Med. 2025 Sep 29;14(19):6905. doi: 10.3390/jcm14196905 (PMC12524352; doi:10.3390/jcm14196905)
Supplement: Supplementary file 1 [file jcm-14-06905-s001.zip › jcm-3850104-supplementary.pdf]

Table S1. Univariable Cox regression analyses of progression-free survival showing non-significant results within each platinum sensitivity group (In sensitive/partially sensitive groups, patients receiving regimens administered to ≥10% of the group were included).

	Platinum-sensitive (n=170)			Partially sensitive (n=116)			Platinum-resistant (n=121)		
**Variable**	**HR**	**95% CI**	***p* value**	**HR**	**95% CI**	***p* value**	**HR**	**95% CI**	***p* value**
Age at diagnosis (years)	1.01	0.99-1.03	0.196	1.002	0.98-1.02	0.810	0.99	0.97-1.01	0.209
Year of diagnosis									
2003-2014	Ref			Ref			Ref		
2015-2017	1.003	0.67-1.50	0.990	0.78	0.48-1.27	0.323	1.05	0.67-1.63	0.839
2018-2020	0.996	0.67-1.48	0.983	0.83	0.53-1.32	0.431	0.71	0.46-1.12	0.139
Initial FIGO Stage									
Early (I-II)	Ref			Ref			Ref		
Advanced (III-IV)	0.81	0.51-1.29	0.379	1.81	0.58-5.73	0.310	1.87	0.67-5.19	0.229
Primary treatment									
Primary debulking surgery	Ref			Ref			-		
Interval debulking surgery	1.05	0.74-1.48	0.795	1.03	0.70-1.53	0.873	-		
No surgery	5.06	0.69-37.21	0.111	2.32	0.83-6.53	0.110	-		
Primary cytoreduction extent*									
No residual	Ref			Ref			Ref		
Optimal (residual tumor <1 cm)	0.997	0.67-1.49	0.990	1.22	0.75-1.97	0.422	1.15	0.74-1.78	0.542

Suboptimal (residual tumor ≥1 cm)	1.28	0.79-2.08	0.321	1.19	0.72—1.97	0.503	1.45	0.89-2.37	0.134
Unknown	0.98	0.42-2.28	0.954	1.71	0.61-4.80	0.311	0.63	0.15-2.60	0.519
Second-line maintenance									
None	-			-			Ref		
Bevacizumab	-			-			0.72	0.18-2.91	0.641
PARP inhibitors	-			-			-		

Abbreviations: CI, confidence interval; FIGO, International Federation of Gynecology and Obstetrics; HR, hazard ratio; PARP, poly(adenosine diphosphate-ribose) polymerase. *Only patients undergoing primary or interval debulking surgery during primary treatment were analyzed.

Table S2. Univariable and multivariable Cox regression analyses of progression-free survival for platinum-sensitive patients who received taxane plus platinum or pegylated liposomal doxorubicin (PLD) plus carboplatin as second-line chemotherapy.

	Univariable		Multivariable	
**Variable**	**HR (95% CI)**	***p* value**	**HR (95% CI)**	***p* value**
Chemotherapy regimen				
Taxane + platinum[†]	Reference		Reference	
PLD + carboplatin	1.99 (1.34-2.96)	<0.001	1.67 (1.10-2.53)	0.015*
Second-line maintenance				
None	Reference		Reference	
Bevacizumab	0.42 (0.28-0.62)	<0.001	0.47 (0.31-0.70)	<0.001*
PARP inhibitors	0.34 (0.20-0.57)	<0.001	0.34 (0.20-0.57)	<0.001*
PFI (months)	0.995 (0.99-1.003)	0.250	-	
Age at diagnosis (years)	1.01 (0.99-1.03)	0.197	-	
Year of diagnosis				
2003-2014	Reference			
2015-2017	1.003 (0.67-1.50)	0.990	-	
2018-2020	0.996 (0.67-1.48)	0.983	-	
Initial FIGO Stage				
Early (I-II)	Reference			
Advanced (III-IV)	0.81 (0.51-1.29)	0.379	-	
Primary treatment				
Primary debulking surgery	Reference			
Interval debulking surgery	1.05 (0.74-1.48)	0.795	-	
No surgery	5.06 (0.69-37.21)	0.111	-	
Primary cytoreduction extent[‡]				

No residual	Reference		
Optimal (residual tumor <1 cm)	0.997 (0.67-1.49)	0.990	-
Suboptimal (residual tumor ≥1 cm)	1.28 (0.79-2.08)	0.321	-
Unknown	0.98 (0.42-2.28)	0.954	-
First-line maintenance			
None	Reference		
Bevacizumab	1.19 (0.66-2.16)	0.564	-
PARP inhibitors	1.04 (0.33-3.26)	0.952	-
Paclitaxel	0.86 (0.12-6.16)	0.879	-

Abbreviations: CI, confidence interval; FIGO, International Federation of Gynecology and Obstetrics; HR, hazard ratio; PARP, poly(adenosine diphosphate-ribose) polymerase; PFI, platinum-free interval; PLD, pegylated liposomal doxorubicin. *Significantly different. †Taxane refers to either paclitaxel or docetaxel, while platinum refers to either carboplatin or cisplatin. ‡Only patients undergoing primary or interval debulking surgery during primary treatment were analyzed.
